# Effects of Lifelong Low Social Status on Inflammatory Markers in Adult Female Macaques

**DOI:** 10.3390/biom16010159

**Published:** 2026-01-16

**Authors:** Mar M. Sanchez, Kaitlyn Love, Alex van Schoor, Kelly Bailey, Trina Jonesteller, Jocelyne Bachevalier, Maria C. Alvarado, Kelly F. Ethun, Mark E. Wilson, Jessica Raper

**Affiliations:** 1 Emory National Primate Research Center, Emory University, Atlanta, GA 30329, USA; mmsanch@emory.edu (M.M.S.); kaitlyn.farley.love@emory.edu (K.L.); alexvanschoor@gmail.com (A.v.S.); kelly.l.bailey@emory.edu (K.B.); tjone24@emory.edu (T.J.); jbachev@emory.edu (J.B.); malvara@emory.edu (M.C.A.); kelly.f.ethun@emory.edu (K.F.E.);; 2 Department of Psychiatry and Behavioral Sciences, School of Medicine, Emory University, Atlanta, GA 30322, USA; 3 Department of Pathology and Laboratory Medicine, School of Medicine, Emory University, Atlanta, GA 30322, USA; 4 Department of Pediatrics, School of Medicine, Emory University, Atlanta, GA 30329, USA; 5 Marcus Autism Center, Atlanta, GA 30329, USA; 6 Children’s Healthcare of Atlanta, Atlanta, GA 30329, USA

**Keywords:** social stress, nonhuman primate, LPS-stimulation, cytokines

## Abstract

Low social status leads to chronic social stress that predicts risk for physical and mental illness, especially when it starts early in life. To examine the longitudinal effects of low social status on the immune system, this study assessed the effects of low social status on developmental secretory patterns of pro- and anti-inflammatory markers under baseline conditions, as well as in response to an immune challenge (lipopolysaccharide (LPS)-induced activation of pro- and anti-inflammatory cytokines) in a translational rhesus monkey model of lifelong social subordination stress. Baseline blood samples were collected in 27 socially housed female rhesus monkeys (13 dominants, DOM, and 14 subordinates, SUB) during infancy (6 months), the juvenile pre-pubertal period (16 months), and adulthood (9–10 years) to examine the longitudinal effects of social status on inflammatory markers in unstimulated versus LPS-stimulated conditions mimicking exposure to bacterial infection. Basal levels of the stress hormone cortisol in blood were measured to examine associations between inflammation and activity of the hypothalamic–pituitary–adrenal (HPA) axis throughout the life span. Basal peripheral levels of inflammatory markers (e.g., IL-6) increased across development in both SUB and DOM animals with no significant differences. Basal cortisol levels were significantly higher in infancy as compared to adulthood, but no significant effects of social rank were detected. However, in adulthood, SUB animals showed a cytokine-specific immune response to ex vivo LPS stimulation with significantly higher secretions of IL-1β, IL-2, and IL-10 compared to DOM animals, whereas IL-8 response to LPS was lower in SUB animals than in DOMs. This cytokine-specific response to an immune challenge that mimics bacterial infection could reflect dysregulated immune cells that may have short-term adaptation, but at the cost of longer-term risks for low-grade chronic inflammation and accelerated immune aging for socially subordinate female macaques.

## 1. Introduction

Psychosocial stressors activate the cortico-limbic neurocircuits that regulate the glucocorticoid stress hormone (e.g., cortisol secretions) and inflammatory (e.g., cytokine) responses to manage energy resources and ultimately restore homeostasis [1]. Although an acute stress response is critical for survival to adapt to environmental threats or fight infections, chronic activation of the stress system can be damaging to physical and mental health. Indeed, chronic psychosocial stress has been implicated in the susceptibility and progression of a number of adverse health outcomes in humans, including mood disorders (e.g., anxiety and depression), drug addiction, obesity, cardiovascular disease, rheumatoid arthritis, infections, and wound healing, as well as neurodegenerative diseases [2,3].

Social subordination in female macaques has been shown to be a potent and chronic psychosocial stressor [4,5,6] that has helped investigate the long-term impact of chronic psychosocial stress. Rhesus macaque societies are organized in a strict linear matrilineal hierarchy where infants assume their mothers’ social status [7]. Dominant females maintain these hierarchies through direct aggression and harassment of low social status females, leading to reduced affiliation, emotional dysregulation, higher stress responses, and higher expression of inflammatory genes among subordinate females [5,6,8,9,10]. Social subordination begins early in life, with infants of low social status mothers receiving more aggression and secreting elevated cortisol as compared to high social status infants [11,12]. Given that macaque societies are matrilineal, only female offspring remain in these social groups throughout their lives, which creates an opportunity to examine the long-term impact of chronic psychosocial stress from low social status in a nonhuman primate model with translational relevance to humans.

Exposure to acute physical or psychosocial stressors activate the hypothalamic–pituitary–adrenal axis (HPA-axis), which releases highly catabolic stress hormones—e.g., cortisol [1]. Glucocorticoid-mediated negative feedback at the level of the hypothalamus, pituitary, and cortico-limbic circuits shuts down HPA-axis activation to restore homeostasis, but chronic psychosocial stress can overwhelm the system, resulting in HPA axis dysregulation. The rich literature on the long-term effects of social subordination in female macaques shows clear indices of chronic stress and HPA-axis dysregulation in both rhesus and cynomolgus macaques, including enlarged adrenal gland sizes [13,14] and impaired HPA-axis negative feedback [14,15,16,17,18,19], despite inconsistent reports on baseline cortisol levels. Thus, chronic stress from social subordination results in elevated basal cortisol levels in juvenile rhesus monkeys [11,20], whereas inconsistent results are reported in adult macaques. Some studies found higher cortisol levels in subordinate than dominant females [21,22], whereas others found the opposite effect [18,23] or no difference between ranks [24,25,26,27]. These results leave many unanswered questions about the effects of lifelong social subordination on HPA-axis function and other stress-related phenotypes, such as elevated inflammation, and require longitudinal studies to track when these effects emerge during development and how they change across the lifespan.

Mounting evidence suggests that the risk of developing illness from chronic inflammation can be traced back to early development and psychosocial stress [28]. Increased levels of pro-inflammatory cytokines appear to be a part of normal aging in humans and macaques [29,30,31,32,33,34], but psychosocial stress and environmental exposures can amplify inflammatory responses with age, contributing to immune senescence and inflammaging [3,35]. Accelerated aging has been seen in macaque models of early life stress/adversity (ELS/ELA) and social subordination, including accelerated DNA methylation age and shortened telomere length [36], along with long-term alterations in neurochemistry, altered N-acetylaspartic acid (NAA) concentrations [37] and enlargement of brain regions regulating socioemotional process [38], as well as higher expression of inflammatory genes [6,39]. Taken together these data suggest that chronic psychosocial stress, particularly when it starts early in life, may increase risk of chronic inflammation and accelerated aging.

The current study sought to examine the longitudinal impact of chronic psychosocial stress via social subordination on immune function and HPA-axis activity from infancy to adulthood. Specifically, we focused on proinflammatory cytokines and cortisol levels circulating in peripheral blood. We collected blood samples during infancy, peri-puberty, and in adulthood to examine basal (unstimulated) cytokine and cortisol levels, as well as cytokine response to an immune challenge (stimulated). Specifically, the ex vivo whole blood LPS-stimulation assay provides a standardized method for eliciting innate immune activation, allowing the assessment of cytokine reactivity and stress-related differences in innate immune responsiveness [40]. We hypothesized that chronic psychosocial stress would result in sustained elevations in basal secretion of cortisol and pro-inflammatory cytokines (e.g., IL-6), as well as in potentiated release of pro-inflammatory cytokines in response to an immune challenge (LPS stimulation) in socially subordinate females compared to dominant female macaques.

## 2. Methods

2a. Animals

**Figure 1:**
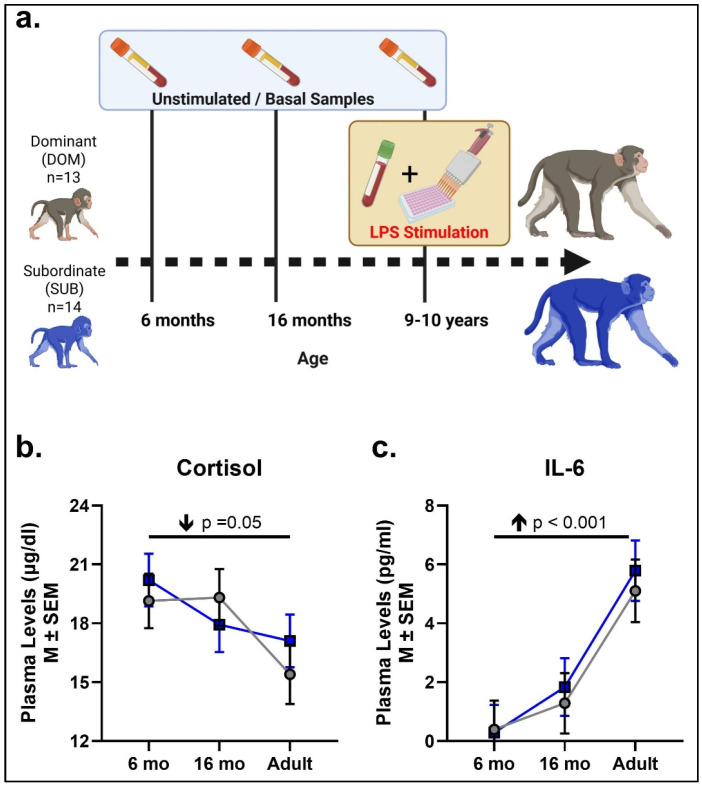
Timeline and longitudinal basal cytokine and cortisol levels across ages. Schematic of the timeline of longitudinal sample collections, created using BioRender.com (**a**). Basal levels of cortisol (**b**) and IL-6 (**c**) were measured in plasma during infancy (6 months), the juvenile period (16 months), and adulthood (9–10 years). SUB females are represented by blue squares and lines, and DOM females by gray circles and lines. Arrows indicate a significant age effect and directionality.

Subjects were 27 female Indian rhesus macaques (*Macaca mulatta*) born at the Emory National Primate Research Center (ENPRC) field station (Lawrenceville, GA) and housed in large indoor/outdoor specific pathogen-free social groups (negative for Herpes B, SIV, SRV, and STLV1). Thirteen females were born to high social ranking dams (dominant—DOM) and 14 were born to dams of low social rank (SUB; Figure 1a). At birth, subjects were assigned to one of the following different postnatal diets: they had access to either a low-calorie diet (LCD) or both a high-calorie diet (HCD) and an LCD (“Choice” diet condition). After menarche, all subjects were assigned to the LCD-only diet condition. The animals were studied longitudinally across infancy (6 months), the juvenile pre-pubertal period (16 months), and adulthood (9–10 years) to examine effects of social status/rank on basal cortisol and inflammatory markers as well as cytokine responses to LPS-stimulation that mimic exposure to bacterial infection. The focus on females for a longitudinal study is more natural for rhesus macaques considering the matrilineal structure of rhesus macaque society. It also provides an important step in identifying underlying mechanisms between social subordination stress and long-term immune function, which can be further explored in male rhesus macaques in the future. All procedures in this study were approved by the ENPRC and the Emory University Institutional Animal Care and Use Committee and were conducted in an AAALAC-accredited facility in full compliance with the United States Public Health Service Policy on Humane Care and use of Laboratory Animals.

2b. Blood sample collection for baseline cortisol and cytokine levels

All animals were trained to quickly separate from their social groupmates for unanesthetized blood collections within 10 min of initial group disturbance, when experimenters first entered the social group. Animals were trained to separate from their social group, come into an indoor cage, transfer to a box, then into a modified housing cage with a squeeze mechanism and an opening for the animal to voluntarily present a leg for an awake blood sample collection through saphenous venipuncture [41,42]. Restraint was only required for the samples collected during infancy, but not during adolescence or adulthood, as the animals were habituated and would readily present their leg for blood collection. Blood samples to examine basal secretions of cortisol and proinflammatory cytokine levels were collected in pre-chilled 2 mL vacutainer tubes containing EDTA (3.6 mg). Samples were centrifuged at 2500 RCF for 15 min in a refrigerated centrifuge (at 4 °C), and plasma was pipetted into sterile cryovials and stored at −80 °C until assayed. During infancy, samples were collected from all 27 animals; two samples were missing during the juvenile period (1 DOM, 1 SUB), and four during adulthood (2 DOM, 2 SUB).

2c. LPS stimulation using whole blood culture

During adulthood, at 9–10 years of age, an ex vivo lipopolysaccharide (LPS) stimulation procedure was used to examine the cytokine response in 23 of the females (DOM = 11; SUB = 12). The protocol for whole blood LPS stimulation was graciously shared by Dr. Heidi Hope at Confluence Discovery Technologies, Inc. (St Louis, MO, USA) [43,44] and piloted in our lab prior to this study to verify the ex vivo elevation of cytokines in response to LPS doses. Specifically, blood samples were collected at room temperature in 4 mL vacutainer tubes containing sodium heparin (75 USP units). The lids of the sodium heparin tubes were opened briefly to introduce oxygen to the blood samples before transport to the lab and stored at room temperature with gentle rocking; then, ex vivo LPS stimulation was performed within 24 h after collection. LPS from Escherichia coli O55:B5 (catalog # L2880, Sigma-Aldrich, Rockville, MD, USA) was dissolved in media solution (consisting of Gibco Dulbecco’s Modified Eagle Medium, high glucose (catalog # 11965, Fisher Scientific, Pittsburgh, PA, USA) with 10% fetal bovine serum (catalog # A5209401, Fisher Scientific) and 1% penicillin-streptomycin-glutamine (catalog # 10-378-016, Fisher Scientific). To standardize the volume incubated, every well contained 160 μL of blood with 20 μL of media solution and 20 μL of LPS or media solution. Two concentrations of LPS 10 ng/mL and 100 ng/mL were tested along with unstimulated control wells containing medium solution, resulting in 24 wells per animal. Blood culture plates were incubated for 5 h in a humidified atmosphere at 37 °C and 5% CO_2_, then centrifuged for 10 min at 10,000 RCF. Plasma was collected and stored at −80 °C until assayed for cytokines.

2d. Plasma cortisol and cytokine assays

Plasma concentrations of cortisol were measured by liquid chromatography–triple quadrupole tandem–mass spectrometry (LC-MS/MS). During infancy and juvenile periods, the LC-MS/MS analyses were performed via reverse-phase chromatography using a Shimadzu Nexera X2 UHPLC system (Shimadzu Scientific Instruments, Canby, OR, USA) coupled with an AB Sciex Triple Quad 6500 Mass Spectrometer (SCIEX, Marlborough, MA, USA) at the Biomarkers Core Laboratory at the ENPRC. During adulthood, LC-MS/MS analyses were performed using Shimadzu Nexera-LCMS-8050 and Shimadzu Nexera-LCMS-8060 instruments at the Endocrine Technologies Core (ETC) at Oregon National Primate Research Center (ONPRC). Plasma cytokine concentrations at all ages were assayed by the Emory Multiplexed Immunoassay Core (Emory University, Atlanta, GA, USA) using a multiplex electrochemiluminescence immunoassay kit from Meso Scale Discovery (Rockville, MD, USA) following the manufacturer’s protocols. EDTA plasma from unstimulated blood samples were assayed for interleukin-6 (IL-6) using V-PLEX NHP IL-6 kit (Catalog #K156QXD), Meso Scale Discovery (Rockville, MD, USA). Plasma from ex vivo whole blood LPS stimulation was assayed using the V-PLEX Proinflammatory Panel 1 (Catalog # K15056D), which included measures for interferon gamma (IFN-γ) and interleukins-1B, -2, -6, -8, and -10. Several samples had below-detectable levels and were missing from analyses, specifically 10 samples for IFN-γ and two samples for IL-2; all other cytokines were in the detectable range.

2e. Statistical analyses

A general linear model (GLM) was used to analyze the longitudinal unstimulated (basal) blood concentrations for IL-6 and cortisol, with age (6 mo, 16 mo, adulthood) as a repeated measure and social rank (DOM, SUB) as fixed factors, using diet (LCD, choice) as a covariate. To control for potential differences in cortisol levels due to differences in LC-MS/MS instrumentation, z-scores were derived from plasma cortisol concentrations and re-analyzed using GLM as previously described. Results did not differ between the raw and z-scored plasma cortisol levels; therefore, the graphs and analyses reports provided here represent the raw cortisol values. Z-score values and results will be provided upon request. Whole blood LPS stimulation data were analyzed for each cytokine separately using GLM with LPS level (unstimulated/0, 10, 100 ng/mL) as a repeated measure, social rank (DOM, SUB) as fixed factors, and diet (LCD, choice) entered as a covariate in the models. For the cytokine response to LPS, we applied the Benjamini–Hochberg method to correct for multiple comparisons, controlling the False Discovery Rate (FDR) at 5%. Results were graphed using GraphPad Prism 10.5 (GraphPad Software), and cytokine data was analyzed using SPSS 31 for Windows (IBM), significance was set at *p* < 0.05, and effect sizes were calculated using ηp2.

## 3. Results

3a. Basal (unstimulated) plasma cortisol levels

Blood concentrations of the stress hormone cortisol significantly decreased with age from infancy (6 months) to adulthood (main age effect: F[2, 70] = 3.13, *p* =0.05, ηp2 = 0.08; Figure 1b), with no significant effects of social rank (F[1, 70] = 0.15, *p* = 0.69, ηp2 = 0.002) or Age X Social Rank interactions detected.

3b. Basal (unstimulated) plasma cytokine levels

IL-6 plasma concentrations increased significantly with age, from 6 and 16 months of age to adulthood (main age effect: F[2, 68] = 13.92, *p* < 0.001, ηp2 = 0.29; Figure 1c). DOM and SUB females did not differ in their IL-6 basal levels (Social Rank: F[1, 68] = 0.20, *p* = 0.66, ηp2 = 0.003). No significant Age X Social Rank interactions were detected, either.

3c. LPS-stimulated cytokine response (adulthood)

**Figure 2:**
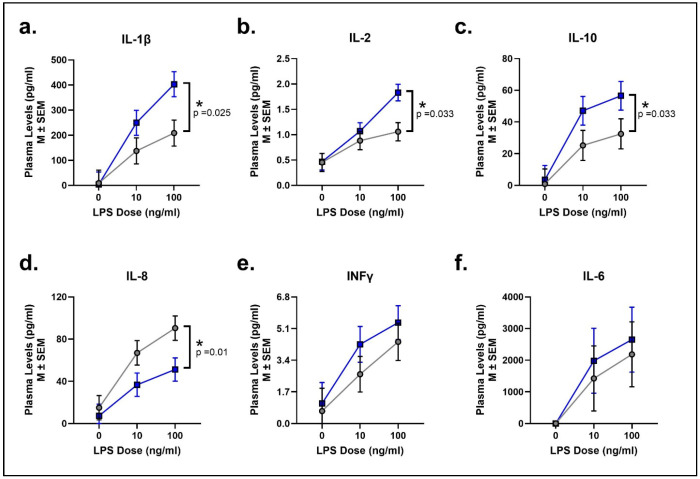
Social subordination alters the cytokine response profile to an immune challenge. Ex vivo whole blood LPS stimulation resulted in dose-dependent cytokine responses in adult female rhesus macaques. SUB females (blue squares and lines) exhibited significantly higher IL-1β (**a**), IL-2 (**b**), and IL-10 (**c**) plasma secretion compared to DOM females (gray circles and lines). SUB females also showed significantly lower IL-8 responses (**d**) compared to DOM females. No differences were detected between groups for INFγ (**e**) or IL-6 (**f**). * indicates a significant effect of social rank.

Adult female rhesus macaques exhibited a dose-dependent increase in cytokine release in response to immune LPS stimulation of whole blood. More importantly, social rank directly affected the inflammatory response to LPS exposure. There was a significant effect of social rank, such that SUB adult females exhibited a significantly higher IL-1β, IL2, and IL10 release response than DOM animals (Social Rank: F[1, 62] = 5.13, *p* = 0.025, ηp2 = 0.08; F[1, 59] = 4.73, *p* = 0.033, ηp2 = 0.07; F[1, 62] = 4.77, *p* = 0.033, ηp2 = 0.07, respectively; Figure 2a–c). This higher IL-1β, IL-2, and IL-10 release suggests an exaggerated inflammatory response to LPS challenge. In contrast, SUB females had a significantly lower IL-8 response as compared to DOM females (Social Rank: F[1, 62] = 7.16, *p* = 0.01, ηp2 = 0.10; Figure 2d), suggesting that chronic stress from social subordination may lead to a diminished immune response to fight off infection. There were no social rank effects for INF-y or IL-6 (F[1, 52] = 1.21, *p* = 0.28, ηp2 = 0.02; F[1, 62] = 1.50, *p* = 0.23, ηp2 = 0.02, respectively; Figure 2e,f). Among the six cytokines tested, four remained significant after applying the Benjamini–Hochberg method, indicating a controlled FDR of 5% for these cytokine responses to LPS stimulation.

## 4. Discussion

This study sought to examine the longitudinal effects of low social status (i.e., social subordination) on immune function from infancy to adulthood and its relationship with basal stress neuroendocrine activity. To achieve this, we studied its effects on developmental secretory patterns of pro- and anti-inflammatory markers under baseline conditions, as well as in response to an immune challenge that mimics exposure to a bacterial infection (LPS-induced activation) in a translational rhesus monkey model of lifelong social subordination stress. We hypothesized that lifelong exposure to chronic psychosocial stress would result in elevated basal peripheral cortisol and proinflammatory cytokine levels, as well as a potentiated proinflammatory cytokine response to LPS stimulation in socially subordinate females compared to dominant female macaques. We found that basal cortisol levels were significantly higher in infancy as compared to adulthood, but without significant effects of social rank. Basal levels of peripheral proinflammatory marker IL-6 increased across development in both SUB and DOM animals without significant differences between the groups. The effects of low social status were more apparent in stimulated compared to unstimulated samples. Thus, in adulthood, SUB animals showed a cytokine-specific immune response to stimulation with a whole blood LPS challenge, with significantly higher secretions of IL-1β, IL-2, and IL-10 compared to DOM animals, whereas IL-8 secretion in response to LPS was lower in SUB than in DOM animals. This cytokine-specific response to an immune challenge that mimics bacterial infection could reflect dysregulated immune cells that may have short-term adaptation, but long-term costs such as social stress become chronic and lifelong [3]. Whether this response to LPS constitutes a risk for low-grade chronic inflammation and accelerated aging for subordinate animals is currently unknown.

Chronic stress activation of the HPA-axis results in elevated secretion of glucocorticoid stress hormones such as cortisol [1]. Although glucocorticoid-mediated negative feedback is meant to shut down prolonged HPA-axis activation to restore homeostasis, chronic psychosocial stress can overwhelm the system, resulting in HPA-axis dysregulation. Social subordination in female macaques is a potent and chronic psychosocial stressor [4,5,6] that has contributed to the understanding of the long-term impact of social determinants of human health. Social subordination in rhesus macaques begins early in life, with low social status females receiving more aggression and evidence of elevated cortisol levels already during adolescence [11,20]. The extensive literature on the long-term effects of social subordination in adult female macaques shows clear indications of chronic stress and HPA-axis dysregulation in both rhesus and cynomolgus macaques, including enlarged adrenal gland sizes [13,14] and impaired HPA-axis negative feedback [14,15,16,17,18,19], despite inconsistent reports on baseline cortisol levels. Some studies have found higher cortisol levels in SUB than DOM females [21,22], whereas other studies found the opposite effect [18,23], and many other studies have found no difference in basal cortisol levels between social ranks [24,25,26,27]. Our study attempted to contribute some answers to the inconsistent findings in the literature by using a longitudinal, within-subjects design to examine the emergence of chronic HPA activation during development and how these effects change across the lifespan. Our study not only confirms a previous finding that basal cortisol decreased with age from infancy to 3 years of age [45], but also extends this finding by showing that the decline in basal HPA-axis activity is present across the lifespan until adulthood (9–10 years old), making a unique contribution to the field. However, no differences in basal cortisol were found between socially dominant and subordinate females at any age point. As suggested in previous reports, the effects of social subordination may be more apparent in response to challenges that stimulate the HPA-axis, including acute stressors and pharmacological challenges to the pituitary and adrenal glands, or that test glucocorticoid-mediated negative feedback [5,11,18,20], which we did not examine in this study.

Inflammation is an essential function of the immune system that protects the host from bacteria, toxins, and infections by eliminating pathogens and promoting wound healing and recovery [3]. This immune response is characterized by a temporary increase in inflammation that occurs when a threat is present and resolves once the threat has passed. However, psychological and environmental stressors can also elicit a systemic inflammatory response [1,2,3]. In adult macaques, social subordination is a potent psychosocial stressor, such that low social status results in higher stress responses and higher expression of inflammatory genes among subordinate females [5,6,8,9,10]. Although the current study did not find a difference in basal (unstimulated) IL-6 levels between subordinate and dominant female macaques, both groups exhibited an increase in IL-6 levels from infancy and peri-puberty to adulthood. Similar effects of increasing IL-6 levels with age have been seen in humans and animals, comparing young adults to individuals of advanced age [3,30,31,32,33]. To our knowledge, this is the first demonstration of an increase in basal IL-6 during an earlier developmental period (from infancy to peri-puberty). Given that the adult females in our study were young adults at the time of collection, it will be important to continue to monitor basal IL-6 levels as they age.

Bacterial challenges (e.g., LPS, *Staphylococcus enterotoxin B*) to whole blood or PMBCs (peripheral blood mononuclear cells) are powerful ex vivo assays to probe immune function. The current study revealed a robust cytokine response was dose-dependent with increasing levels of LPS, not driven by single marginal *p*-values. More importantly, social status influenced the production of IL-1β, IL2, IL-10, and IL-8 in response to LPS stimulation. Specifically, social subordination results in an exaggerated cytokine response with low social status females producing significantly more IL-1β, IL-2, and IL-10, but lower IL-8 secretion in response to LPS than dominant females. These findings parallel previous studies showing higher immune defense and inflammatory gene expression in socially subordinate female macaques [6,10]. Considering its anti-inflammatory properties, increased IL-10 may reflect counter-regulatory feedback to overall increased inflammation from LPS stimulation. Although an exaggerated IL-10 response may be interpreted as compensatory regulation, we are proposing that it could also be an early sign of inflammaging. For example, older adults exhibit a greater IL-1β in response to LPS stimulation [46,47] and elevated IL-10 response to bacterial challenges have been seen in aged humans, macaques, and mice [32,48,49,50]. This exaggerated IL-1β and IL-10 response to LPS among subordinate female macaques may suggest that chronic psychosocial stress from low social rank poses a risk for accelerated aging. Age-related increases in inflammation are also evident in immune cell populations in rhesus macaques living on the island of Cayo Santiago, Puerto Rico [51]. Future studies should examine both basal levels of IL-1β and IL-10, as well as immune cell type, to confirm the possibility of inflammaging in socially subordinate female macaques.

Subordinate female macaques also secreted lower levels of IL-8 in response to the LPS stimulation compared to females with high social status. IL-8 (also known as CXCL8) plays an important role in stimulating the immune response to threats by activating and recruiting neutrophils to the site of infection, delaying cell apoptosis, and promoting the production of reactive oxygen species [52]. Thus, low IL-8 production could suggest that chronic psychosocial stress (via social subordination) leads to reduced immune response to challenge. A previous study found that subordinate rhesus macaques had decreased T-cell production [53], which could result in a weakened immune response to pathogens and increased susceptibility to disease. Conversely, higher expression of genes involved in viral defense is found in high-ranking female macaques [10], which further supports the important influence of social rank on immune function. Indeed, human patients with inflammatory conditions (e.g., surgery or infection) exhibit reduced IL-8 production to LPS stimulation compared to healthy controls [54,55]. However, in the absence of an inflammatory condition, healthy aged adults exhibit exaggerated production of IL-8 in response to bacterial challenges compared to young adults [56,57,58]. Further studies are needed to investigate how chronic psychosocial stress from social subordination influences the inflammatory response to an immune challenge.

The production of INF-γ and IL-6 by whole blood LPS stimulation did not differ between females of different social statuses. Interestingly, previous studies of whole blood LPS stimulation found that IL-6 production differed between young-adult (5–16 year-old) and aged (>20 year-old) nonhuman primates [33,49]. Considering that females in this study would fall into the ‘young adult’ age category, it is possible that social status-dependent differences in IL-6 response to LPS stimulation may yet emerge at an older age.

In addition to stress-induced alterations in HPA-axis function, socially subordinate rhesus females also show alterations in their hypothalamic–pituitary–gonadal (HPG) axis, resulting in disruptions of ovarian function and reproductive endocrinology that included reductions in sex steroid levels—particularly estradiol and progesterone [5,13,17,20,59]. Given the powerful role of sex steroids on immune function, which has been the focus of recent studies (see [60] for a review), one cannot rule out a contribution of social subordination-induced differences in gonadal hormones on the immune effects detected in our study. Unfortunately, the current study does not have concurrent gonadal hormone data to examine this possibility. Thus, our study cannot disentangle the direct effect of psychosocial stress from potential indirect effects on the reproductive axis that alter cytokines, overall immune function, and health in socially subordinate animals (e.g., [5,17,61]); however, gonadal hormones should be considered in future research.

The current study has several limitations that should be considered when interpreting the results. First, there were instrumentation differences between the LC-MS/MS assays of cortisol levels between early and late age points. However, performing a z-score correction revealed the same age effect with no differences for social rank. Although the current sample size is large for a nonhuman primate study, the significant *p*-values with low to moderate effect sizes suggest that a larger number of females per social rank would be necessary to detect interactions between factors. The early life diet (choice, low-calorie diet) manipulation is an important early life exposure with potential confounding long-term effects on the current study. To control for this confound, we used diet as a covariate in our statistical analyses, which further limited our statistical power to detect significant effects due to the sample size. Lastly, this study was unable to examine sex as a biological variable because it only included female macaques. A focus on females for a longitudinal study is more natural for rhesus macaques considering the matrilineal structure of rhesus macaque society. It also provides an important first step in establishing potential measurable links between social subordination stress and long-term immune function, thus opening the door for future studies to include males. Future studies on males would need to consider how social rank changes between transitions from natal group, to adolescent group, to novel breeding group in adulthood [7].

## 5. Conclusions

In summary, chronic psychosocial stress from low social status greatly influences immune functions across the lifespan. Neither basal circulating levels of proinflammatory cytokines nor cortisol differed by social status, although differences in the HPA axis could emerge under challenge conditions, which were not tested in this study. The present study found robust social rank differences in cytokine-specific responses to an immune challenge (LPS stimulation) in adulthood. The exaggerated cytokine response to immune challenge in adult subordinate females may reflect immune cell dysregulation due to social stress and could also pose a risk for low-grade chronic inflammation and accelerated aging. Future studies should concentrate on both cytokine levels and immune cell populations to determine whether social subordination increases the risk of inflammaging.

## Data Availability

The raw data supporting the conclusions of this article will be made available by the authors on request.
